# Second Life for Plastic Fibre Waste Difficult to Recover: Partial Replacement of the Binder in Asphalt Concrete Mixtures by Dry Incorporation

**DOI:** 10.3390/ma16030948

**Published:** 2023-01-19

**Authors:** Mireia Ballester-Ramos, Helena Miera-Dominguez, Pedro Lastra-González, Daniel Castro-Fresno

**Affiliations:** 1 BECSA SAU Edificio Simetría, Paseo Buenavista s/n, 12100 El Grao de Castellón, Spain; 2 GITECO Research Group, Universidad de Cantabria, Avda. de Los Castros s/n, 39005 Santander, Spain

**Keywords:** asphalt mixture, plastic fibre waste, dry process, binder replacement

## Abstract

In previous studies, different additives and modifiers have been studied to improve the properties of asphalt concrete mixtures, whose main failures are plastic deformation and cracking. In this research, the improvement of the properties of asphalt concrete mixtures were investigated by introducing residual plastics as a substitute for virgin bitumen, which improves the sustainability of the mixtures. Furthermore, the results obtained from these new mixtures were compared with a mixture designed with polymer-modified bitumen (PMB). Ten experimental designs were tested with three types of waste fibre plastics from a municipal solid waste treatment plant and two percentages of bitumen replacement (15% and 25%). The experimental testing plan included air void characterization, moisture sensitivity, stiffness and fatigue resistance, among others. An increase of approximately 5% in voids could be observed when introducing the plastic material and therefore some tests were carried out to over-compact the specimens. The results showed an improvement in the mechanical performance of the experimental mixtures, highlighting the resistance against plastic deformations, which even reached similar values to the mixtures made with PMB.

## 1. Introduction

In recent times, there has been a growing awareness that the development of societies should be as sustainable as possible. The road sector is not oblivious to this demand and is making augmented efforts to develop products and processes to increase the contribution of road infrastructures to sustainability. In particular, in recent years, there has been a lot of research aimed at reducing the environmental impact of asphalt mixtures for flexible pavements, not only from the point of view of reducing the carbon footprint of their vital process, but also by turning them into active tools that recycle by-products or waste material. The ultimate goal is to design asphalt mixtures with similar or superior technical performance to conventional asphalt mixtures, but with a lower environmental impact.

One of the most used asphalt mixtures is asphalt concrete (AC) because it can handle high traffic loads. However, because there is an increase in the demand for higher and different load configurations each year, as well as constant temperature changes caused by global warming, this mixture is prone to failures of various kinds, such as plastic deformation and cracking. To solve these problems, different additives or modifiers have been studied to improve the durability of AC mixtures, such as polymer modified bitumen (PMB) [1,2,3], fibres [4,5], plastics [6,7], etc. However, these additives also have disadvantages: the main drawbacks are the cost, which turns out to be high; the increase in mixing temperature, which leads to higher environmental pollution; and the problems of segregation between the asphalt-polymer phases during transport to the paving site or during static storage at high temperature [1].

At the same time, the consumption of polymer products has increased worldwide in recent decades from 230 million tons in 2009 [8] to 368 million tons in 2019 [9], an increase of 60% in only 10 years. This trend has resulted in a large waste stream that must be properly managed to avoid environmental damage. For this reason, this article focuses on residual plastics as an additive in asphalt mixtures, especially recycled plastic from municipal solid waste treatment plants, which could be used to promote a circular and sustainable economy and thus reduce production costs. This kind of facility combines automatic and manual sorting processes for the separation of recoverable fractions from the municipal solid waste mixture. In the first stage, bulky waste is separated manually. Subsequently, different automatic processes are applied to separate and classify the waste according to composition, colour, material, etc. Once the residual fractions have been separated, they are subjected to a mechanical process consisting of crushing, washing, centrifuging and drying.

In order to integrate residual plastic samples in bituminous mixtures, it is necessary to subject them to a grinding process to obtain particle size fractions of 2/10 mm, which is achieved by means of a knife mill. Once shredded, these fractions are washed in order to remove debris and other residues that may have adhered to them, as well as other elements such as labels or stickers. For this washing, water and different concentrations of surfactants or surfactants with a high soda content are used at a temperature ranging from 25 °C to 40–70 °C. Any traces of surfactants and soda that may have adhered to the particles are removed by successive rinses with clear water. In addition, there are alternative cleaning routes which allow the reduction in organic surface contamination that do not involve the use of water, and are based on dry cleaning, by friction of the surface between the plastic particles. The last pre-treatment step consists of drying the cleaned particles to achieve complete removal of moisture. This treatment has been used to obtain the plastics that will be analysed in this article.

Once the plastics have been prepared for inclusion in the mixture, there are two ways of incorporating them into it: a wet or dry process. The first one consists of crushing the plastics and adding them to the hot bitumen; subsequently, the plastic-modified bitumen is added to the mixer as a binder. This is the most common method, but the principal disadvantages are the necessity for specialized plants for the digestion process at high temperatures, expensive costs and compatibility problems [10,11,12]. In addition, the maximum amount of plastic that can be added to the mixture is smaller (8%) than by the dry method (more than 15%) [13], reducing the impact on the recycling rate. The second one consists of adding the plastics directly into the mixer together with the aggregates and bitumen. This technique is less developed than the wet method [14,15,16,17,18]; however, it is easier to apply since it is not necessary to make major modifications to the asphalt plant and requires less energy. This facilitates the spread of the process and facilitates the reuse of polymeric waste. Furthermore, it is more economically profitable [19] and allows the incorporation of larger quantities of plastics (greater than 15%) [13].

Until now, the impact has been mostly assessed with conventional bitumen only. In this case, the study includes new properties analysed, such as cracking energies, and the extent to which they reach the performance of PMB. All the information provided so far leads to the study carried out by this research, which consists of the design of new asphalt mixtures incorporating polymeric wastes using the dry method. This work evaluated the suitability of different residual plastics to replace virgin bitumen by analysing the mechanical behaviour of AC mixtures in which a binder fraction has been replaced by three different types of plastic fibre waste, whose cost is lower than other polymeric materials.

## 2. Materials and Methodology

### 2.1. Materials

The materials used are those normally employed in the manufacture of asphalt mixtures, except for plastic fibre waste of difficult valorisation. Ophite, a type of porphyry igneous rock commonly employed in the north of Spain, was used in the coarse fraction, while limestone was used in the fine fraction and filler. A conventional 50/70 penetration grade bituminous binder and a commercially available polymer-modified bitumen (PMB 45/80-65) were used to manufacture the mixtures. The properties of the aggregates and bitumen used are shown in Table 1 and Table 2, respectively.

Plastic fibre waste materials have to meet the technical requirements of homogeneity and cleanliness. The former is a key point because the properties and composition of the plastic waste should be approximately the same over time, in order to achieve a similar behaviour, independently of the moment the asphalt mixture is manufactured. This point is difficult to achieve because some plastic waste packages depend highly on the season (summer, Christmas, etc.). Cleanliness is another key issue, in order to avoid adding organic materials to the mixer. Apart from fulfilling these conditions, it is necessary that enough plastic waste is generated so that big quantities can be used in road manufacturing.

**Figure 1:**
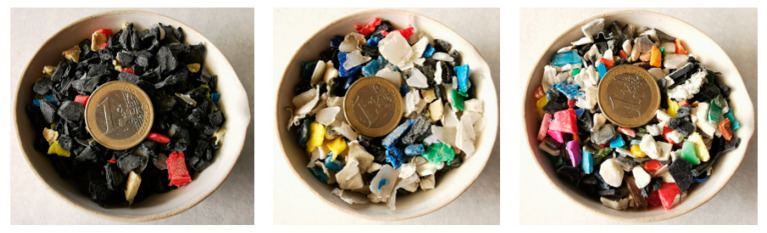
Plastic waste. From left to right: PLA-1, PLA-2 and PLA-3.

The selected plastic waste of this project fulfils these technical requirements. They were segregated fractions in the municipal solid waste treatment plant of Algimia (Castellón, Spain) managed by the company “Residuos Palancia Belcaire” (Figure 1). The theoretical composition and recovery technique applied to each one is detailed below:

- PLASTIC-1 (PLA-1) is a mixture of baskets. This fraction is separated manually at the bulky waste triage stage.
- PLASTIC-2 (PLA-2) is a mixture of drums, pipes, toys, etc. This fraction is separated manually at the bulky waste triage stage.
- PLASTIC-3 (PLA-3) is a PP/PE polymer blend obtained as a residual fraction from a solid waste sorting process by means of optical sorting.

**Figure 2:**
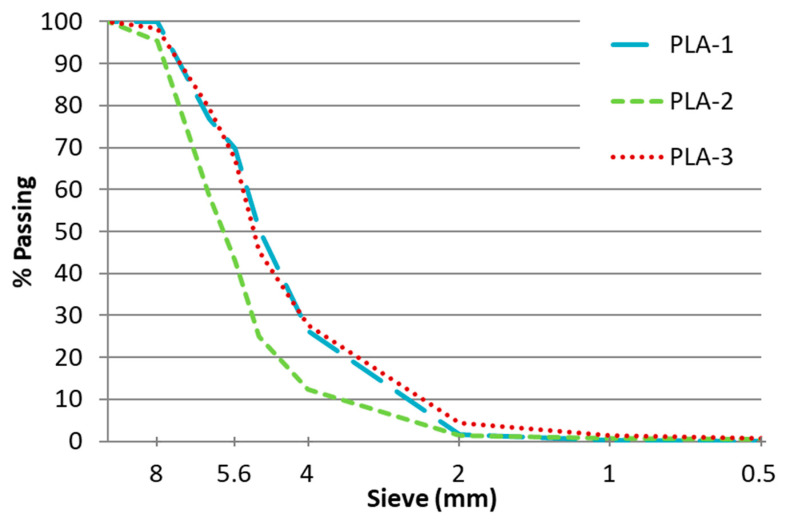
Granulometry of the analysed residual plastic.

Different technical tests were carried out to characterize the plastics used. Figure 2 represents the granulometry (UNE-EN 933-2) of each residual plastic. It can be seen that 8 mm was the maximum size and the particle distribution was quite similar, especially between PLA-1 and PLA-3, which had an almost identical granulometry.

The exact composition of the residual plastics was calculated using infrared spectrophotometry (FT-IR) and differential scanning calorimetry (DSC) according to EN ISO 11357-1 and EN ISO 11357-3 standards, respectively. In addition, the density was obtained through the ISO 1183-1 standard, method B: liquid pycnometer. The components and density of each sample were as follows:

- PLA-1: Blend of low-density polyethylene (LDPE), medium-density polyethylene (MDPE), ethylene-vinyl acetate copolymer (EVA), and polypropylene (PP) in a minor quantity, with the presence of SiO_2_ and CaCO_3_. This plastic had a density of 0.902 ± 0.030 g/cm^3^.
- PLA-2: High density polyethylene (HDPE) with the presence of SiO_2_ and CaCO_3_. Density was 0.879 ± 0.021 g/cm^3^.
- PLA-3: Polypropylene (PP), medium-density polyethylene (MDPE) and low-density polyethylene (LDPE) with the presence of SiO_2_ and CaCO_3_. In that case, the plastic’s density was 0.936 ± 0.009 g/cm^3^.

### 2.2. Sample Preparation

The manufacturing temperature was determined according to the information provided by the bitumen supplier; therefore, reference and experimental mixtures were produced at the same temperature (150 °C), because they used the same 50/70 penetration grade binder. Only the control mixture, which was produced with PMB 45/80-65 binder, was different; in this case, the manufacturing temperature was 165 °C according to the supplier.

For the preparation of the experimental mixtures, part of the virgin bitumen was replaced with residual plastics. This replacement was made in volume, trying to modify the structure of the reference mixture as little as possible. The incorporation of waste plastic was carried out using the dry process, pouring them directly into the mixing drum at room temperature. This method was selected because even though the plastics are joined with the hot material, they are not pre-heated, which minimizes the generation of gases [15]. In addition, this technique is a simple alternative that has been shown to be effective when making experimental mixtures, and it can be easily replicated in practically any asphalt plant. Two possible methods were studied within the options presented by the dry process:

- Method A: Incorporation of residual plastics in the coarse fraction. The plastic was poured over the coarse fraction before incorporating the rest of the aggregates and bitumen, so that the plastics soften forming “bonds” with this type of aggregate.
- Method B: Incorporation of waste plastics after pouring bitumen. When bitumen was added to the coarse and fine aggregate fraction, it formed a film around them, so that the plastics were mostly embedded in the matrix which forms the mortar of the mix.

**Figure 3:**
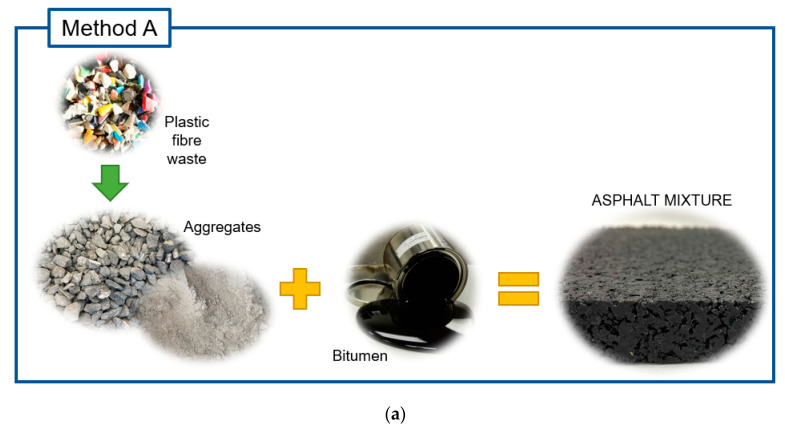

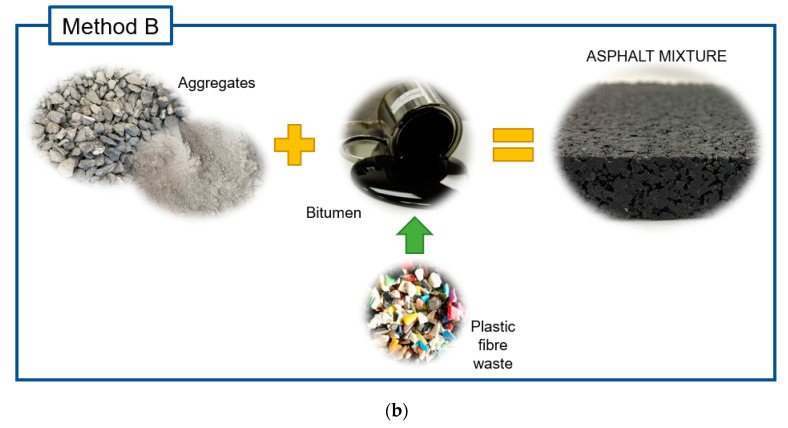
Methods for adding residual plastics to dry mix processing. (**a**) plastic over aggregates (**b**) plastic over bitumen.

**Figure 4:**
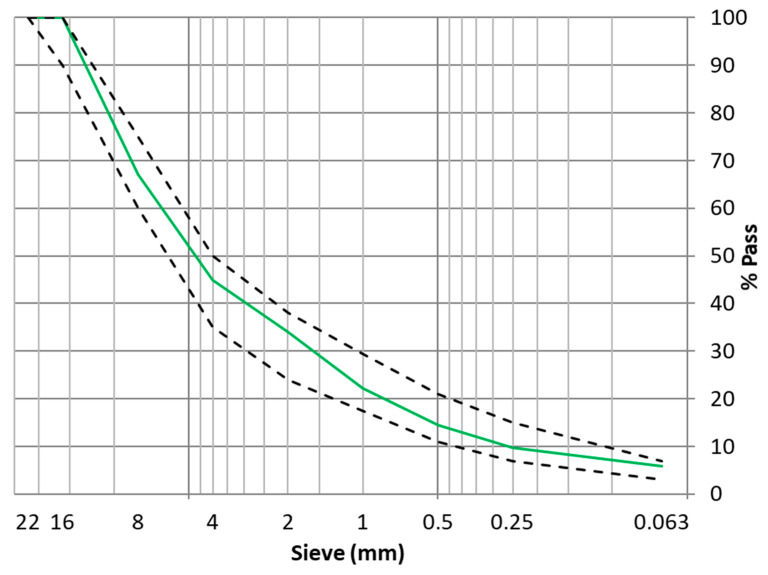
Particle size distribution in AC mixture.

A scheme of the two methods can be seen in Figure 3. The mixing time was increased for one minute at the time of incorporating the residual plastics to ensure a correct dispersion of the residual plastic in the mixture, which was checked visually. In addition, the particle size distribution was the same in all mixtures (Figure 4), varying only the amounts of bitumen and residual plastics used.

### 2.3. Mixture Designs 

Ten experimental asphalt concrete (AC) mixtures were developed for this work. The first 6 designs were produced by varying the type of waste plastic (PLA-1, PLA-2 and PLA-3) and the method of incorporation (method A and B). While the last 4 experimental mixtures were produced by raising the percentage of bitumen replaced and varying the type of compaction (normal or over-compacted). The nomenclature of the different designs was defined based on the type of plastic, the type of incorporation method and compaction. Thus, the designed mixture PLA1-A-N corresponds to the AC mixture with PLA-1 manufactured using method A and normal compaction. Similarly, the mixture PLA2-B-OC corresponds to the AC mixture with PLA-2 manufactured using method B and compacted with twice as many conventional blows. Table 3 details the different designs carried out.

Experimental mixtures were compared with a reference and a control AC mixture for surface layer, whose difference was the type of bitumen used in their manufacture. Both mixtures had a void content of approximately 5%. This characteristic was achieved with a bitumen content of 4.3% by weight of mixture in both cases. A double comparison was conducted to analyse how much the experimental mixtures improve with respect to a conventional bitumen and, at the same time, to check if this improvement was comparable with that obtained using PMB. 

### 2.4. Experimental Work Plan

The first milestone of this research was to obtain a residual plastic that provided the greatest functional improvements to the asphalt mixture, as well as the most efficient manufacturing method. To achieve this milestone, the results of the following tests were evaluated: air void content (EN 12697-8), the Marshall test (EN 12697-34), the water sensitivity test (EN 12697-12) and the wheel tracking test (EN 12697-22). All of them were performed on experimental mixtures containing 15% virgin bitumen replaced with waste plastics, always incorporated using the dry process. 

The second milestone of the research was more ambitious and consisted of optimizing the virgin bitumen content that could be replaced, while maintaining the feasibility of manufacturing asphalt mixtures and the mechanical behaviour. To do so, the percentage of virgin bitumen replaced with plastic waste was increased to a maximum percentage of 25%. During this process, it was found that the impact of plastics was particularly significant on the density of the mixtures, increasing the percentage of voids, so it was decided to use two different compaction energies for the final design of the experimental mixtures. The first one was a normal compaction, which means that the same energy was applied as was applied to the reference mixtures. On the other hand, the energy used for the over-compacted specimens was twice that used in the reference mixtures. 

To accomplish the second milestone, in addition to the mechanical test mentioned before and in order to check the cohesion of the experimental mixtures due to their high percentage of voids, the Cantabro test (EN 12697-17) was also performed on them.

Finally, to fully characterize the asphalt mixtures, stiffness (EN 12697-26) and fatigue resistance (EN 12697-24) were evaluated to check the dynamic performance of the over-compacted mixtures which showed the best balanced.

#### 2.4.1. Air Voids and Marshall Tests

To measure bulk density and air voids in accordance with the European EN 12697-8 standard, Marshall specimens were used, compacted by 75 blows on each side in accordance with the European EN 12697-30 standard. It should be noted that the over-compacted specimens received 150 blows per face. Subsequently, the Marshall test was performed even though it is not currently included in the Spanish regulations, since it is one of the tests that has historically been most used to design bituminous concretes. Four replicates were performed for each mixture.

#### 2.4.2. Water Sensitivity Test

The purpose of the water sensitivity test (EN 12697-12) is to determine the loss of cohesion caused by saturation and the action of water on a bituminous mixture. To do so, 8 cylindrical specimens were manufactured, compacted at 50 blows per face, except for the over-compacted ones that received 100 blows per side, which were divided into two batches of equal size. While one batch was left in dry conditions, the other was submerged in water for 3 days at 40 °C before it was broken. The indirect tensile strength (ITS) was determined in both dry and wet conditions (ITS_dry_ and ITS_wet_) and the moisture susceptibility was obtained and expressed as a percentage according to Equation (1).

(1) $$\mathrm{ITSR}\;\left(\%\right)=\frac{{\mathrm{ITS}}_{\mathrm{wet}}}{{\mathrm{ITS}}_{\mathrm{dry}}}\times 100$$

#### 2.4.3. Cracking Energy Test

**Figure 5:**
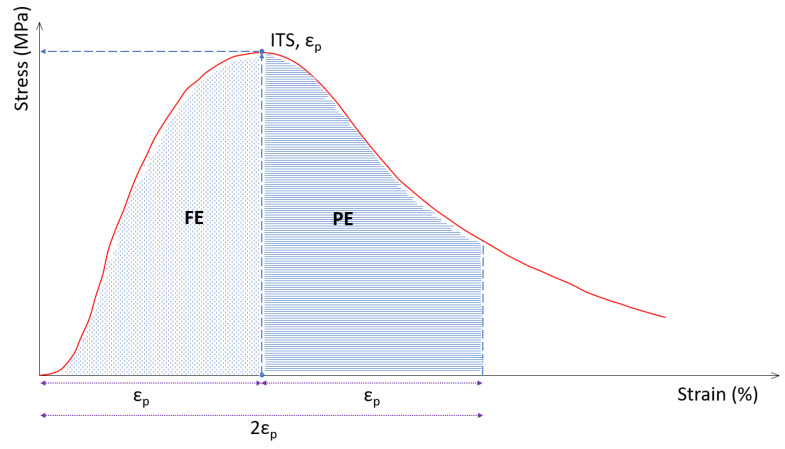
Stress–strain curve recorded from ITS.

For the measurement of toughness, many researchers agree that the indirect tensile test is the most suitable test due to its simplicity [20]. Stress–strain curves were recorded when the indirect tensile strength was determined (EN 12697-23). The fracture energy (FE) and post-cracking energy (PE) were calculated as the area under the curve before and after peak stress was reached, respectively, as shown in Figure 5. The former (FE) was considered representative of the cracking resistance while the second (PE) showed the resistance against cracking propagation [21,22]. Cracking toughness was measured as the sum of both parameters. The performance of the mixtures was analysed in dry and wet conditions, so the impact of water damage was also assessed in relation to the propagation of fissures.

#### 2.4.4. Wheel Tracking Test

To evaluate the rutting resistance of the experimental mixtures in accordance with the European standard EN 12697-22, the wheel tracking test was used. In this analysis, two prismatic specimens per mixture were made with the dimensions 410 mm × 260 mm × 50 mm. Conditioning and testing were carried out at a temperature of 60 °C. In this case, the result was determined by the slope, calculated using Equation (2).

(2) $${\mathrm{WTS}}_{\mathrm{aire}}=\frac{d_{10,000}-d_{5000}}{5}$$

where WTS was the inclination of the wheel track in mm for 10^3^ loading cycles, and d_5000_, d_10,000_ were the bearing depth after 5000 and 10,000 load cycles in mm.

#### 2.4.5. Cantabro Particle Loss Test

This test is traditionally applied for the evaluation of particle loss in porous asphalt mixtures. In this study, it was used on over-compacted mixtures as a method to check the cohesion of the mixtures, since a high percentage of voids could be considered as a possible risk. In accordance with the EN 12697-17 standard, the specimens were subjected to abrasion in the Los Angeles machine to measure the particle loss obtained after 300 turns. This loss is expressed as a percentage and is calculated by Equation (3).

(3) $$\mathrm{Particle}\;\mathrm{loss}\;\left(\%\right)=\frac{m_{i}-m_{f}}{m_{i}}\times 100$$

where m_i_ and m_f_ were the initial and final mass of the specimens.

#### 2.4.6. Stiffness and Fatigue Resistance Tests

These tests are key to evaluating the performance of the pavement as they condition the transmission of loads and its service life in the passage of axles. Moreover, they must be analysed together since the stiffness of a bituminous mixture is directly related to the deformation it undergoes and, therefore, to its fatigue damage. 

**Figure 6:**
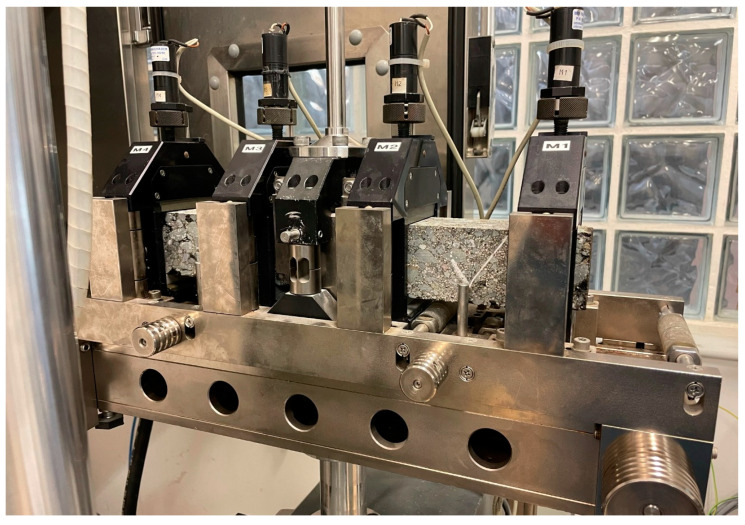
Fatigue resistance test.

Following the EN 12697-26 (Annex B) standard, a stiffness modulus analysis was carried out by means of the four-point bending test (Figure 6). In the case of fatigue strength, the EN 12697-24 (Annex D) standard and the four-point bending test were used.

For these tests, eight specimens of each mixture with dimensions of 410 mm × 60 mm × 60 mm were required, which were obtained by cutting a specimen of 80 mm in height. Both tests were performed at 20 °C. The stiffness test was carried out in controlled deformation mode with a deformation amplitude of 50 µm/m at different frequencies, from 0.1 Hz to 30 Hz. The fatigue test was carried out by applying a frequency of 30 Hz in controlled deformation mode. The main test parameters obtained from this test were the fatigue law calculated through Equation (4) and the deformation at one million cycles.

(4) $$\varepsilon \;\left(m/m\right)=C_{1}\cdot 10^{-3}\cdot N^{-C_{2}}$$

where N is the number of loading cycles for a given level of strain ε (m/m); C_1_ and C_2_ are the fatigue constants.

#### 2.4.7. Statistical Analysis

The results obtained from the experimental mixtures were statistically analysed using Minitab software to determine if the differences with the REF and CONTROL mixtures were significant. To do this, first, the normality of the data and the homogeneity of the variances were checked through the Kolmogorov–Smirnov and the Levene statistical test, respectively. Depending on the results obtained, Student’s *t*-test was used when a normal distribution of results and homogeneity of variances were observed, and the Mann–Whitney U-test was used otherwise. For all cases, a 95% confidence interval (*p*-value 0.05) and a significance level of 5% were considered. 

## 3. Results and Discussion

### 3.1. Selection of the Suitable Materials and Manufacturing Method

The results of the first phase to select the most suitable materials are presented in Table 4. The general trend was towards increased voids regardless of the type of plastic and the mixing process used, as in previous studies [7]. Since the substitution of bitumen with the waste plastics was conducted by volume, this increase in voids was associated with a reduction in the workability of the mix. The viscosity difference between the hot bitumen and the residual plastic when incorporated into the mixing drum were considered to be the main reason for the increase. Increasing the temperature is not a solution because the bitumen could be aged and the plastics could produce fumes harmful to human health [15]. In addition, the increase in temperature will raise the energy consumed to produce the mixtures which would impair sustainability.

Nevertheless, in spite of the significant increase in voids, the experimental mixtures with residual plastics show a similar stability to the reference mixture with no statistical differences between them (Table 5). The deformation depended on the incorporation method and the type of plastic used, not following a uniform criterion; for example, PLA-2 does not show significant differences, while PLA-1 improves with method-A and PLA-3 with method-B. However, these differences were only statistically significant in the case of the PLA3-A-N mixture.

The results of the water sensitivity test (Table 4) show, in general terms and considering the difference in voids, a good performance against water damage of the experimental mixtures. In general, ITSR higher than 85% is considered a good result of the resistance against water damage, but it is also important to study the resistance of each mixture separately. The experimental mixtures did not show statistically significant differences with the reference and control mixtures in dry conditions; despite the higher percentage of voids, only the plastic waste PLA-1 showed a significant decrease in its resistance in wet conditions, independently from the method applied to incorporate it. PLA-2 and PLA-3 showed similar performances to the reference, despite the decrease in bitumen and the increase in the percentage of voids. The experimental mixture did not reach the resistances of the control mixture in wet conditions, as can be seen in Table 5; however, this can be considered coherent due to the behaviour of the PMB. Analysing both methods to add the plastic waste, A or B, the second method achieves higher resistances in general terms. It seems that increasing the contact with the asphalt improves adherence; probably, when the plastic waste is included in the mortar, a better digestion with the asphalt is achieved, while contact between the plastic waste and aggregate does not reach the same adherence. 

The increase in resistance to plastic deformation (Table 4) was very significant in all the experimental mixtures, regardless of the incorporation method. All of them showed exceptionally good values against rutting, especially considering their high percentage of voids. This is reflected in Table 5, where it can be seen that all experimental mixtures show significant statistical differences with respect to the reference mixture. This behaviour could be due to the lower susceptibility of plastics to temperature, forming a stiffer matrix than the REF mixture, even though some of the experimental mixtures do not have statistically significant differences with the CONTROL mixture, as can be seen in Table 5. Therefore, this effect was also related to the greater difficulty in compacting the experimental mixtures.

As a result, in order to continue with the project, PLA-2 and PLA-3 together with the method B were selected to add the plastic waste because they obtained the best performance. This choice of incorporation method differs from previous studies [7] where plastics had a better performance when adding them directly over the aggregates. At this point and with these conditions, it could be concluded that the behaviour of the experimental mixtures with plastic waste are similar or even better than the REF mixture, but they do not reach the same level as the CONTROL mixture.

### 3.2. Optimization of the Final Mixture Design

Once the materials and the manufacturing method were chosen, it was decided to increase the virgin bitumen replacement to 25% and apply two compaction energies in order to decrease the percentage of voids: the conventional one and the double compaction energy. Following this, the properties of the experimental mixtures are presented, analysing each test separately.

#### 3.2.1. Air Voids and Marshall Tests

Table 6 presents the results of the void and Marshall tests. As in the previous case, the percentage of voids was significantly higher than the REF and CONTROL mixtures (Table 7 presents the *p*-values), although the impact of increasing the plastic waste was not equal, because the mixture PLA2-B-N showed similar results to the experimental mixtures with 15% of PLA-2 waste material. Furthermore, by doubling the compaction energy, the percentage of voids was decreased by about 1% in both cases. 

As before, the mixtures showed high stability and moderate deformation despite the high percentage of voids, especially when higher compaction energy was applied. In this case, stability and deformation improved significantly, increasing stability significantly and maintaining the deformation of the reference mixture, and achieving statistically similar performance to the control mixture (Table 7).

#### 3.2.2. Water Sensitivity Test

**Figure 7:**
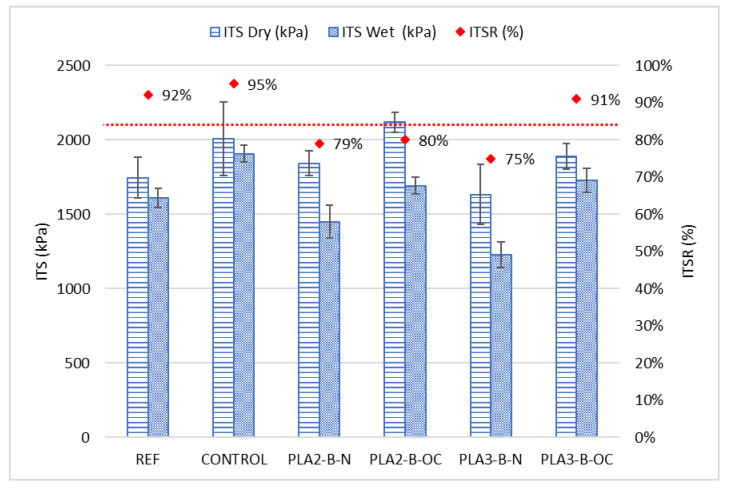
Indirect tensile strength and moisture sensitivity results.

The increase in the percentage of waste plastics, in the case of conventional compaction energy, reduced the resistance in wet conditions below the values obtained for the reference mixture (Figure 7). However, by over-compacting the mixtures, a significant improvement was obtained, reaching better values than the reference mixture in dry conditions and a similar performance in wet (Table 8 presents the *p*-values). Considering the results of the mixtures compacted with the conventional energy, although only the mixture PLA3-B-N had a statistical lower resistance in wet conditions, the general trend of the results indicates that over-compacting the experimental mixtures is highly recommended if a replacement of 25% of virgin binder has to be applied, because their performance is significantly improved. 

On the one hand, both over-compacted experimental mixtures (PLA2-B-OC and PLA3-B-OC) showed high resistances between the reference and the control mixture, even higher than the CONTROL mixture with the PMB binder in the case of the PLA2-B-OC mixture. This leads to a lower ITSR in the case of this mixture, although it was not considered a problematic behaviour because of the good performance in dry conditions and the acceptable value.

On the other hand, mixtures with conventional compaction had low values of tensile strength in wet conditions, so this property should be carefully controlled just in case conventional compaction is applied.

#### 3.2.3. Cracking Energy Test 

**Figure 8:**
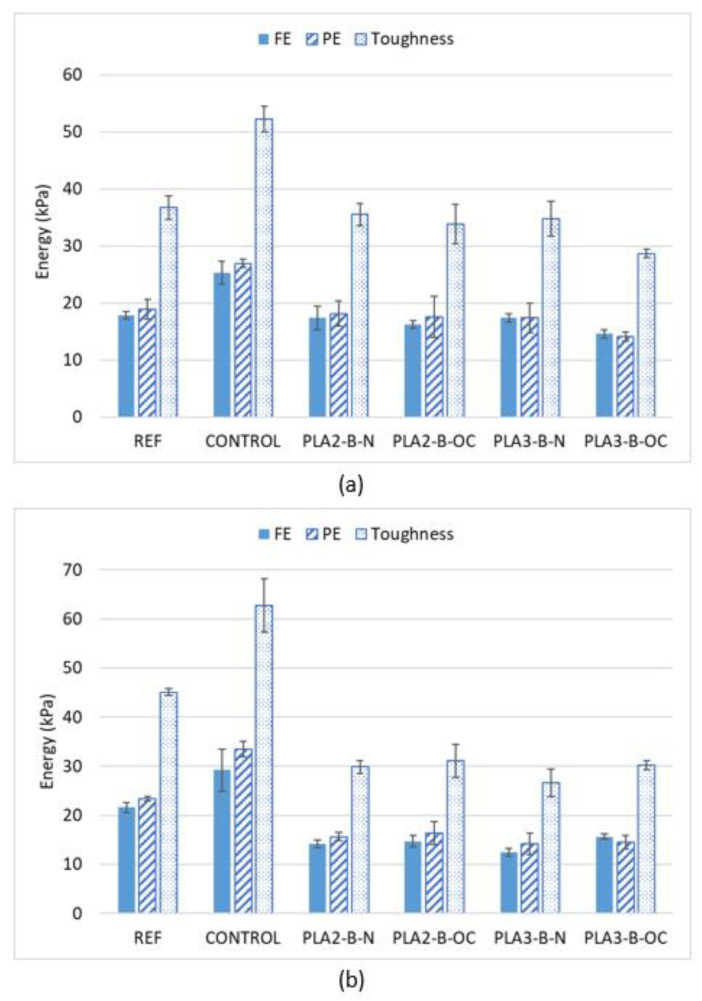
Fracture properties. Dry conditions (**a**); wet conditions (**b**).

The mean values of each parameter (FE, PE and toughness) are shown in Figure 8 in dry and wet conditions in order to check the impact of the plastic waste on cracking resistance. 

**Figure 9:**
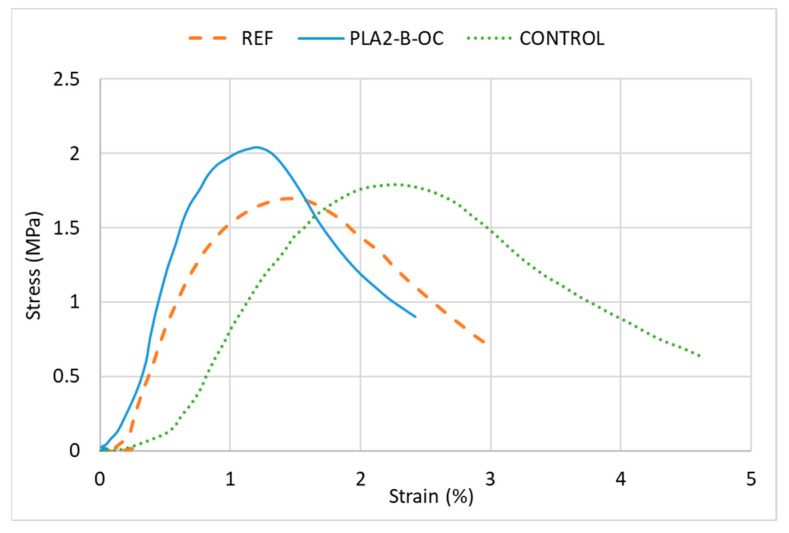
Stress–strain curves for three example specimens.

According to the results, the resistance against cracking of the experimental mixtures is far from the control mixture with PMB binder. The energies when the plastic waste is incorporated are in all cases statically lower than the CONTROL mixture (Table 9 presents the *p*-values). This makes sense if we considered the higher elasticity and ductility of the PMB in relation to the conventional penetration grade binder, which has a clear impact on the fracture and post-cracking energies of the asphalt mixtures, even more when a significant percentage of this bitumen has been replaced with plastic waste, which is clearly stiffer. This could be explained through Figure 9, which shows an example of three specimens (REF, experimental and CONTROL), where it can be appreciated that the strength of the experimental specimen is higher while the strain is lower than in the case of the REF and CONTROL mixtures; therefore, lower energy is obtained.

In comparison with the REF mixture, which has the same type of binder, the behaviour of the experimental mixtures can be divided depending on the test conditions: in dry conditions, the performance is similar, although, curiously, the over-compacted mixtures obtained a lower FE while the performance of the conventional compacted mixtures was better. The decrease in the FE in wet conditions was higher, so it seems the fracture properties could be worsened by the replacement of the virgin bitumen with plastic waste, especially in the case of waste material PLA-3. A deeper analysis is required; as over-compaction does not improve cracking resistance, the maximum percentage of virgin bitumen replaced could be limited to values lower than 25%, at least with these types of plastic waste. 

#### 3.2.4. Wheel Tracking Test

Resistance to plastic deformation is one of the properties most significantly improved by the incorporation of waste plastics (Table 10), as in previous studies [7], which is coherent with the point that plastic waste stiffens the mixture. In this case, the slope obtained by all the experimental mixtures was notably lower than that of the REF mixture; however, it was difficult to analyse the differences between the mixtures with plastic waste since all the values can be considered as optimal, and they move in a range of results similar to the CONTROL mixture (Table 11). The reason for such a good performance, despite the high number of voids, was probably due to the fact that the plastic wastes remain practically unchanged at 60 °C, while the bitumen softens.

#### 3.2.5. Cantabro Particle Loss Test

**Figure 10:**
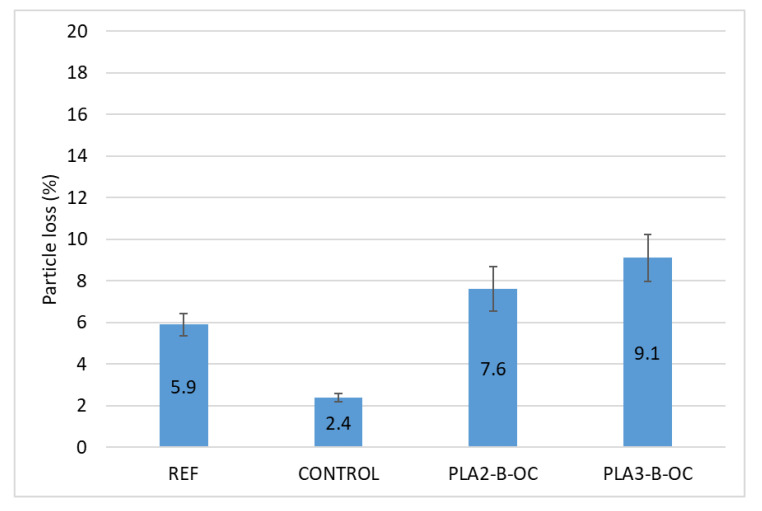
Mean values of Cantabro particle loss test.

Although the results obtained for the experimental mixtures show a loss of particles higher than that obtained for the REF and CONTROL mixtures (Figure 10), in both cases they were far from the limit values proposed for porous mixtures (20% in the most limiting case). The differences between the experimental and conventional mixtures were statistically significant (Table 12). Since this type of failure is not common in this type of mixture, loss of cohesion was not considered to be a problem despite the significant increase in voids.

#### 3.2.6. Stiffness and Fatigue Resistance

For a complete characterization, stiffness and fatigue tests were performed on the over-compacted specimens since these mixtures were considered as the best experimental mixtures. The stiffness is presented in Table 13. The incorporation of residual plastics increased the modulus of the reference mixture, despite the difference in the percentage of voids. This increase is more significant at low frequencies when the conventional mixtures present lower elastic behaviour. These results were consistent with the increase in strength obtained earlier in the track test. The PLA-3 waste material stiffens the reference mixture at a higher level than PLA-2. Furthermore, this behaviour was reinforced by the reduction in the phase angle over the whole frequency range, which implies a higher elastic behaviour in all cases.

The results of the fatigue resistance tests are shown in Table 14. It can be seen that when bitumen was replaced with plastic waste, the experimental mixtures obtained a quite similar fatigue resistance with values close to that of the REF mixture. In addition, the higher modulus of the PLA-3 mixture does not seem to penalize its fatigue resistance.

Therefore, it did not appear that the dynamic properties of the mixtures penalized the use of residual plastics, since their dynamic behaviour was quite similar to the reference mixture, despite the increase in voids and the reduction in virgin bitumen.

## 4. Conclusions

In the present research, the effect of replacing virgin bitumen with different plastic fibre wastes was experimentally evaluated. After analysing different residues, optimizing the addition process and evaluating the mechanical performance, the following conclusions were reached:

- The feasibility of replacing virgin bitumen with plastic waste was demonstrated from a mechanical point of view. The 25% replacement rate is technically feasible as long as the compaction energy is increased to control the increase in voids, although cracking performance should be analysed in more depth.
- Method B was selected to incorporate the plastics into the manufacture of the mixture, which consists of adding the plastics after the bitumen using the dry process, embedding them in the mortar of the bituminous mixture.
- Experimental mixtures showed a significant increase in voids due to differences in the viscosity of residual plastics with respect to virgin bitumen.
- Regarding the residual plastics used, PLA-2 and PLA-3 showed better mechanical results and are considered the priority alternatives from a technical point of view.
- The increase in resistance to plastic deformation is particularly noteworthy. As for the rest of the properties, the experimental mixtures obtained similar or moderately better results than the reference mixture. However, it should be noted that the cracking energy in wet conditions is lower than the energy of the reference mixture.
- Mixtures, despite improving with respect to reference mix, are not as good as those manufactured with commercial polymer-modified bitumen, although in the case of resistance against plastic deformation they are quite similar.

After these conclusions, some properties that have not been assessed are the recyclability of the experimental mixtures, the possible modification of skid resistance and the potential generation of microplastics due to wear caused by vehicles on the roads. Therefore, future lines of research have been opened up, focused on analysing this technology. 

## Figures and Tables

**Table 1:** Properties of aggregates.

Properties	Result	Limits	Standard
Ophite			
Los Angeles coefficient	15	≤20	EN 1097-2
Specific weight (g/cm^3^)	2.794	-	EN 1097-6
Polished Stone value (PSV)	>56	≥50	EN 1097-8
Flakiness Index (%)	8	≤20	EN 933-3
Limestone			
Los Angeles coefficient	28	-	EN 1097-2
Specific weight (g/cm^3^)	2.724	-	EN 1097-6
Sand equivalent	78	>55	EN 933-8

**Table 2:** Properties of bitumen.

Properties	Result	Standard
50/70		
Specific weight (g/cm^3^)	1.035	EN 15326
Penetration at 25 °C	57	EN 1426
Softening point (°C)	51.6	EN 1427
Fraass brittle point (°C)	−11	EN 12593
PMB 45/80-65		
Relative density	1.027	EN 15326
Penetration (0.1 mm)	56	EN 1426
Softening point (°C)	71	EN 1427
Elastic recovery at 25 °C (%)	88	EN 13398
Fraass brittle point (°C)	−15	EN 12593

**Table 3:** Asphalt concrete mixture designs.

ID Nº	Mixture Design	Waste Plastic		Compaction	Bitumen	
		Type	Incorporation Method		Type	% Replaced
1	REF	-	-	Normal (N)	B 50/70	-
2	CONTROL	-	-	N	PMB 45/80-65	-
3	PLA1-A-N	PLA-1	A	N	B 50/70	15
4	PLA1-B-N	PLA-1	B	N	B 50/70	15
5	PLA2-A-N	PLA-2	A	N	B 50/70	15
6	PLA2-B-N	PLA-2	B	N	B 50/70	15
7	PLA3-A-N	PLA-3	A	N	B 50/70	15
8	PLA3-B-N	PLA-3	B	N	B 50/70	15
9	PLA2-B-N	PLA-2	B	N	B 50/70	25
10	PLA2-B-OC	PLA-2	B	Over-compacted (OC)	B 50/70	25
11	PLA3-B-N	PLA-3	B	N	B 50/70	25
12	PLA3-B-OC	PLA-3	B	OC	B 50/70	25

**Table 4:** Mechanical performance. Analysis of the plastic addition process and type of plastics.

Results	REF	CONTROL	PLA1-A-N	PLA1-B-N	PLA2-A-N	PLA2-B-N	PLA3-A-N	PLA3-B-N
Void test (EN 12697-8)								
Binder content (%)	4.3	4.3	3.7	3.7	3.7	3.7	3.7	3.7
Density (g/cm^3^)	2.45 ± 0.01	2.44 ± 0.01	2.32 ± 0.01	2.32 ± 0.01	2.33 ± 0.01	2.34 ± 0.01	2.36 ± 0.01	2.36 ± 0.01
Voids in mixture (%)	5.1 ± 0.4	5.4 ± 0.3	11.2 ± 0.2	11.4 ± 0.4	10.7 ± 0.3	10.4 ± 0.2	9.8 ± 0.3	9.6 ± 0.3
Voids in aggregates (%)	15.3 ± 0.3	15.6 ± 0.3	19.5 ± 0.2	19.7 ± 0.3	19.1 ± 0.3	18.8 ± 0.2	18.2 ± 0.3	18.1 ± 0.2
Marshall test (EN 12697-34)								
Stability (kN)	15.7 ± 0.8	18.1 ± 0.9	15.6 ± 1.5	14.8 ± 0.9	14.5 ± 0.5	14.7 ± 0.9	15.8 ± 1.1	15.8 ± 0.4
Deformation (mm)	3.8 ± 0.3	3.5 ± 0.4	3.3 ± 0.6	4.3 ± 1.3	3.7 ± 0.6	3.9 ± 0.6	4.7 ± 0.6	4.1 ± 0.4
Water sensitivity test (EN 12697-12)								
ITS-Dry (kPa)	1746 ± 138	2006 ± 248	1730 ± 101	1903 ± 144	1939 ± 153	2018 ± 50	1687 ± 147	1756 ± 190
ITS-Wet (kPa)	1610 ± 63	1907 ± 55	1240 ± 62	1428 ± 65	1641 ± 61	1703 ± 85	1577 ± 82	1675 ± 164
ITSR (%)	92	95	72	75	85	84	93	95
Wheel tracking test (EN 12697-22)								
Slope (mm/1000 cycles)	0.08 ± 0.03	0.02 ± 0.00	0.05 ± 0.02	0.02 ± 0.01	0.04 ± 0.01	0.04 ± 0.00	0.03 ± 0.00	0.03 ± 0.01
Rut Depth (mm)	3.1 ± 0.4	1.6 ± 0.4	2.0 ± 0.3	1.5 ± 0.3	2.1 ± 0.4	2.2 ± 0.1	1.8 ± 0.4	1.7 ± 0.3

**Table 5:** *p*-values from experimental tests. Reference and control mixture.

***p*-Values (REF)**	**PLA1-A-N**	**PLA1-B-N**	**PLA2-A-N**	**PLA2-B-N**	**PLA3-A-N**	**PLA3-B-N**
Void test (EN 12697-8)						
Density	0.000	0.030	0.000	0.000	0.000	0.000
Voids in mixture	0.000	0.030	0.000	0.000	0.000	0.000
Voids in aggregates	0.000	0.030	0.000	0.000	0.000	0.000
Marshall test (EN 12697-34)						
Stability	0.665	0.175	0.066	0.146	0.890	0.915
Deformation	0.237	0.555	0.849	0.769	0.046	0.353
Water sensitivity test (EN 12697-12)						
ITS ^Dry^	0.859	0.174	0.120	0.034	0.583	0.932
ITS ^Wet^	0.000	0.010	0.513	0.140	0.544	0.885
Wheel tracking test (EN 12697-22)						
Slope	0.164	0.042	0.074	0.077	0.051	0.060
Rut Depth	0.013	0.005	0.015	0.039	0.006	0.007
***p*-Values ** **(CONTROL)**	**PLA1-A-N**	**PLA1-B-N**	**PLA2-A-N**	**PLA2-B-N**	**PLA3-A-N**	**PLA3-B-N**
Void test (EN 12697-8)						
Density	0.000	0.030	0.000	0.000	0.000	0.000
Voids in mixture	0.000	0.030	0.000	0.000	0.000	0.000
Voids in aggregates	0.000	0.030	0.000	0.000	0.000	0.000
Marshall test (EN 12697-34)						
Stability	0.149	0.003	0.002	0.003	0.025	0.009
Deformation	0.591	0.378	0.659	0.347	0.021	0.134
Water sensitivity test (EN 12697-12)						
ITS ^Dry^	0.112	0.514	0.670	1.000	0.091	0.171
ITS ^Wet^	0.000	0.000	0.001	0.010	0.001	0.030
Wheel tracking test (EN 12697-22)						
Slope	0.063	0.391	0.072	0.001	0.013	0.028
Rut Depth	0.105	0.747	0.102	0.058	0.382	0.530

**Table 6:** Bulk density and air void results.

Results	REF	CONTROL	PLA2-B-N	PLA2-B-OC	PLA3-B-N	PLA3-B-OC
Void test (EN 12697-8)						
Binder content (%)	4.3	4.3	3.2	3.2	3.2	3.2
Density (g/cm^3^)	2.45 ± 0.01	2.44 ± 0.01	2.35 ± 0.01	2.38 ± 0.01	2.32 ± 0.01	2.35 ± 0.01
Voids in mixture (%)	5.1 ± 0.4	5.4 ± 0.3	10.8 ± 0.4	9.8 ± 0.2	11.9 ± 0.3	10.7 ± 0.3
Voids in aggregates (%)	15.3 ± 0.3	15.6 ± 0.3	18.0 ± 0.3	17.1 ± 0.2	19.0 ± 0.3	18.0 ± 0.3
Marshall test (EN 12697-34)						
Stability (kN)	15.7 ± 0.8	18.1 ± 0.9	17.0 ± 0.6	20.9 ± 0.8	14.8 ± 0.6	17.9 ± 0.8
Deformation (mm)	3.8 ± 0.3	3.5 ± 0.4	4.8 ± 0.4	3.9 ± 0.5	4.4 ± 0.4	3.3 ± 0.7

**Table 7:** *p*-values for volumetric properties and Marshall test.

***p*** **-Values (REF)**	**PLA2-B-N**	**PLA2-B-OC**	**PLA3-B-N**	**PLA3-B-OC**
Void test (EN 12697-8)				
Density	0.000	0.000	0.000	0.000
Voids in mixture	0.000	0.000	0.000	0.000
Voids in aggregates	0.000	0.000	0.000	0.000
Marshall test (EN 12697-34)				
Stability	0.053	0.000	0.123	0.014
Deformation	0.013	0.805	0.049	0.265
***p*** **-Values (CONTROL)**	**2-N**	**2-OC**	**3-N**	**3-OC**
Void test (EN 12697-8)				
Density	0.000	0.000	0.000	0.000
Voids in mixture	0.000	0.000	0.000	0.000
Voids in aggregates	0.000	0.000	0.000	0.000
Marshall test (EN 12697-34)				
Stability	0.099	0.005	0.002	0.804
Deformation	0.009	0.350	0.026	0.615

**Table 8:** *p*-values (water sensitivity test).

***p*** **-Values (REF)**	**2-N**	**2-OC**	**3-N**	**3-OC**
ITS ^Dry^	0.295	0.009	0.394	0.139
ITS ^Wet^	0.064	0.114	0.001	0.069
***p*** **-Values (CONTROL)**	**2-N**	**2-OC**	**3-N**	**3-OC**
ITS ^Dry^	0.665	0.596	0.066	0.665
ITS ^Wet^	0.002	0.003	0.000	0.015

**Table 9:** *p*-values for cracking energy results.

***p*** **-Values (REF)**	**PLA2-B-N**	**PLA2-B-OC**	**PLA3-B-N**	**PLA3-B-OC**
FE Dry	0.715	0.064	0.514	0.052
PE Dry	0.638	0.622	0.463	0.046
Toughness Dry	0.473	0.299	0.436	0.022
FE Wet	0.000	0.000	0.000	0.001
PE Wet	0.000	0.010	0.019	0.009
Toughness Wet	0.000	0.004	0.008	0.000
***p*** **-Values (CONTROL)**	**PLA2-B-N**	**PLA2-B-OC**	**PLA3-B-N**	**PLA3-B-OC**
FE Dry	0.003	0.004	0.006	0.030
PE Dry	0.030	0.047	0.025	0.000
Toughness Dry	0.000	0.004	0.004	0.030
FE Wet	0.006	0.007	0.005	0.008
PE Wet	0.000	0.000	0.001	0.000
Toughness Wet	0.001	0.001	0.000	0.001

**Table 10:** Rutting results.

Results	REF	CONTROL	PLA2-B-N	PLA2-B-OC	PLA3-B-N	PLA3-B-OC
Wheel Tracking Test (EN 12697-22)						
Slope (mm/1000 cycles)	0.08 ± 0.03	0.02 ± 0.00	0.02 ± 0.01	0.03 ± 0.01	0.05 ± 0.01	0.04 ± 0.00
Rut Depth (mm)	3.1 ± 0.4	1.6 ± 0.4	1.8 ± 0.3	2.6 ± 0.1	2.4 ± 0.4	2.0 ± 0.2

**Table 11:** *p*-values of rutting results.

***p*** **-Values (REF)**	**PLA2-B-N**	**PLA2-B-OC**	**PLA3-B-N**	**PLA3-B-OC**
Slope (mm/1000 cycles)	0.042	0.056	0.150	0.077
Rut Depth (mm)	0.008	0.113	0.060	0.032
***p*** **-Values (CONTROL)**	**PLA2-B-N**	**PLA2-B-OC**	**PLA3-B-N**	**PLA3-B-OC**
Slope (mm/1000 cycles)	0.391	0.018	0.041	0.001
Rut Depth (mm)	0.304	0.023	0.025	0.073

**Table 12:** *p*-values of particle loss test.

***p*** **-Values (REF)**	**PLA2-B-OC**	**PLA3-B-OC**
Particle loss (%)	0.046	0.007
***p*** **-Values (CONTROL)**	**PLA2-B-OC**	**PLA3-B-OC**
Particle loss (%)	0.052	0.001

**Table 13:** Stiffness test of the over-compacted final dosages.

	REF		CONTROL		PLA2-B-OC		PLA3-B-OC	
Frequency (Hz)	Dynamic Modulus (MPa)	Phase Angle (°)	Dynamic Modulus (MPa)	Phase Angle (°)	Dynamic Modulus (MPa)	Phase Angle (°)	Dynamic Modulus (MPa)	Phase Angle (°)
0.1	657	42.7	1012	29.5	1191	32.5	1682	25.9
0.2	880	41.3	1226	29.4	1457	30.8	2002	26.3
0.5	1299	39.6	1618	29.1	1924	28.8	2540	23.8
1	1712	37.9	2005	28.6	2329	27.3	3021	22.5
2	2227	35.9	2475	27.8	2863	25.8	3590	20.9
5	3095	32.8	3271	26.0	3600	23.5	4347	19.0
10	3916	30.1	4013	24.3	4260	20.6	4992	16.6
20	4830	27.2	4810	22.6	4951	18.9	5717	17.5
30	5516	26.0	5403	22.9	5414	18.1	6200	14.1

**Table 14:** Fatigue strength of over-compacted experimental mixtures and reference mixture.

Mixtures	Initial Modulus (MPa)	Strain-Characteristic * (µm/m)	Fatigue Line	R^2^
REF	4138	181.5	$\varepsilon \;\left(m/m\right)=6.110\cdot 10^{-3}\cdot N^{-0.2545}$	0.95
CONTROL	5403	270.3	$\varepsilon \;\left(m/m\right)=1.185\cdot 10^{-3}\cdot N^{-0.1069}$	0.80
PLA2-B-OC	4610	176.2	$\varepsilon \;\left(m/m\right)=1.433\cdot 10^{-3}\cdot N^{-0.1517}$	0.88
PLA3-B-OC	4308	172.2	$\varepsilon \;\left(m/m\right)=1.880\cdot 10^{-3}\cdot N^{-0.1731}$	0.94

* 10^6^ cycles.

## Data Availability

The data presented in this study are available on request from the corresponding author.
